# Awareness and Knowledge of Caesarean Section Complications Among Women in Jeddah, Saudi Arabia

**DOI:** 10.7759/cureus.32152

**Published:** 2022-12-03

**Authors:** Roaya M Yaqoub, Maryam A Khouj, Afnan A Alsaif, Ghaida A. Eissa, Jood A Alhemdi, Samera Albasri

**Affiliations:** 1 Obstetrics and Gynaecology, Ministry of National Guard - Health Affairs, Jeddah, SAU; 2 Faculty of Medicine, King Abdulaziz University, Jeddah, SAU; 3 Paediatrics, Royal Commission Medical Center, Yunbu, SAU; 4 Obstetrics and Gynaecology, King Abdulaziz University Hospital, Jeddah, SAU

**Keywords:** emergency and elective cesarean, cesarean complications, complications of c-section, attitude of pregnant women, c-section

## Abstract

Objectives: To assess the awareness about and attitude towards the complications of Cesarean section in the antenatal and postnatal period among women in Jeddah, Saudi Arabia.

Methods: This cross-sectional study was conducted between January 2020 and September 2020, involving 507 women in the antenatal and postnatal period in Jeddah, Saudi Arabia. Data were obtained via online surveys. The questionnaire addressed the knowledge about short and long-term complications.

Results: Most participants received a poor knowledge score for the awareness of Cesarean section complications (45.4%), and only 12.6% had good knowledge. Most participants were in the age group of 32-42 years. Most participants were university-educated and had an excellent socioeconomic status. A statistically significant relationship was detected between the age group and the participant's level of knowledge regarding Cesarean sections (P = 0.030) and between the level of knowledge and experiencing Cesarean delivery by maternal request (P = 0.029).

Conclusion: The study concluded that pregnant women had poor awareness regarding the complications of Cesarean sections. Most participants had a negative attitude toward Cesarean deliveries and preferred vaginal delivery.

## Introduction

Cesarean section (C-section) is the most common procedure in obstetrics worldwide [1]. A C-section is a surgical procedure to deliver one or more fetuses via the abdominal wall and uterine incision [2]. According to the World Health Organization (WHO), since 1985, the acceptable percentage of worldwide C-sections has been 10-15% of all deliveries [3]. One of the most critical findings of the American Center for Disease Control and Prevention found that the Cesarean delivery rate has increased from 20.7% in 1995 to 31.6% in 2016 [4].

It is important to mention that Cesarean deliveries are life-saving procedures when there are obstetrical indications. In many rural areas of the world, like many African countries, a high mortality rate was associated with the inability to perform C-sections due to inefficient healthcare systems. On the other hand, it is alarming to know that there has been a significant increase in C-section delivery rate in developed countries, exceeding the safe range, and many are for no apparent medical reason [5,6]. In the Western world, women have only one or two children, while in the East or the Middle East, women are culturally coerced into having many children. Thus, they are more prone to multiple Cesarean deliveries in their lifetime [7,8]. This statement is supported by the results of studies published in 2017 in Egypt and India, which showed that repeated C-sections are the dominant cause of the number of C-sections [9,10]. Like the rest of the countries, Saudi Arabia is facing a rapid rise in Cesarean deliveries. In 2016, the C-sections in Jeddah reached 26.3% [11]. Recent evidence explored the prevalence of Cesarean delivery on maternal request (CDMR) in King Abdulaziz Medical City in Riyadh. It showed that at least 13.7% of C-sections were not due to medical indication [12]. A study published in 2018 in Qassim city concluded that out of the 936 deliveries that occurred in the study duration, most (55.4%) were C-sections, of which 12.2% were elective and 43.3% were due to medical indications [13].

Cesarean delivery indications include fetal malpresentation, multiple pregnancies, chorioamnionitis, arrested labour, oligohydramnios, cord prolapse, cephalo-pelvic disorders, and medical diseases such as eclampsia and HELPP (hemolysis, elevated liver enzymes, and low platelets) syndrome. Furthermore, advanced maternal age and CDMR are other indications for Cesarean delivery [9,10]. When compared with vaginal delivery, this procedure is associated with several risks and complications that may increase the rate of mortality and morbidity of both the mother and the fetus [14,15]. Complications can be short-term or long-term. Short-term complications are related to anaesthesia or surgery. Anaesthesia-related complications include hypoxia, drug overdose, and apnea. The most common surgical complications are wounds, abdominal wall, urinary tract, pelvic and respiratory infection, bleeding, internal organ damage, and thromboembolism. Uterine rupture, adhesions, ectopic pregnancies, infertility, and abnormal placental implantation like placenta previa, placenta accreta, placenta increta, and placenta percreta are a few examples of long-term complications [16,17]. In addition, it is worth mentioning that there is a high mortality risk in pregnant women delivered via C-section [18]. C-section rates have increased significantly worldwide over the last few decades [2]. The most common reasons for C-section on demand without medical indication are fear of vaginal birth, avoiding labour complications such as perineal injury and pelvic organ prolapses, and a previous family history of C-section [12,19].

Moreover, the number of patients undergoing Cesarean delivery for non-obstetric reasons has rapidly increased. This could be due to many factors influencing the patients' decisions, including possible fetal outcomes concerning astrological beliefs in some cultures and the convenience of patients and obstetricians [20]. A study published in Iran in 2012 showed that C-section's financial burden, including duration of hospitalization and drugs used, is significantly higher than that of vaginal delivery [21]. Also, women with public insurance were 40% less likely than women with private insurance to opt for C-sections [22]. It also found that higher income tends to result in a higher C-section rate [23]. In contrast, some pregnant women choose C-sections based on the advice of family members and friends that spontaneous vaginal delivery is painful [24].

To our knowledge, there are limited studies evaluating the recognition of C-section complications among women in Jeddah, Saudi Arabia. The current study aims to assess the awareness of women during the antenatal and postnatal period in Jeddah about complications of C-sections.

## Materials and methods

Study design and setting 

This study adopted a quantitative, descriptive, cross-sectional design. It was carried out at the King Abdulaziz University Hospital in the Western Region of the Kingdom of Saudi Arabia (KSA) over four months (January to September, 2020) among women who were currently pregnant or had delivered in Jeddah. Pregnant women with mental illnesses, severely ill patients, and patients who did not give consent for the study were excluded. King Abdulaziz University Hospital is a tertiary care hospital. It employs professionals with distinct medical qualifications, such as consultants, physicians, and technicians in many specialities with international expertise.

Sample size and sampling procedure 

The sample size calculated for this study was 507 participants. The selection was made using a convenience sampling technique, with a 95% confidence level and a 5% margin of error. The calculations were made using a Raosoft sample size calculator [25].

Data collection instrument 

The data was collected via an online survey, and the participants' responses were recorded anonymously in an electronic Google Forms (Google LLC, Mountain View, California, United States) questionnaire filled out by the conductors of this study. The questionnaire consisted of three sections. Section one was about the respondents' socio-demographic characteristics, including age, education level, income, and occupation. Section two consisted of obstetric history starting with gravidity, parity, abortion, and mode of last delivery. Section three was composed of "yes" or "no" questions and divided into three parts. Part one consisted of two questions and inquired about the participants' previous history of C-sections and their indications. The second part was generated to evaluate the preferred mode of delivery and contained two questions. Part three was allocated to assess the participants' knowledge concerning C-section complications and consisted of 15 questions. It was given a score between 0 and 15. Women who responded "yes" to a question received a score of one, while those who gave a "no" received a score of zero. Women who obtained a score between 0-5 were considered to have poor knowledge, those with scores between 6-10 had fair knowledge, and 11-15 were considered to possess sound knowledge about C-section complications.

The reliability of the questionnaire was assessed by a specialist using a test of internal consistency; Cronbach's alpha was (0.71). The coefficient of variation was determined for each statement to indicate the degree of response variability.

Analysis

Microsoft Excel version 16 (Microsoft Corporation, Redmond, Washington, United States) was used for data entry. Regarding data analysis, data were coded, checked, and entered into IBM SPSS Statistics for Windows, Version 26.0 (Released 2019; IBM Corp., Armonk, New York, United States). Qualitative data consisting of socio-demographic information (age group, level of education, occupation, income, and mode of delivery) and the extent to which expectant mothers were aware and their attitudes towards C-section complications were expressed as frequencies and percentages. Numerical data, including the age and the data obtained from the obstetric history section of the questionnaire (gravidity, parity, abortion), were expressed through measures of central tendency, including means ± standard deviations, modes, ranges, and dispersion. Furthermore, categorical data were compared via Chi-squared test to determine the relationship between the obstetric history and socio-demographic characteristics of the participants (age group, education, occupation, income, and the number of pregnancies) with their modes of last delivery, their attitudes toward C-sections, and level of awareness, with p-value <0.05 indicating a significant relationship between the variables

Research ethics

The Institutional Review Board of King Abdulaziz University Hospital, Jeddah, Saudi Arabia, approved this cross-sectional study of our hospital. All pregnant women were confirmed regarding the study objectives and written informed consent was acquired to complete the questionnaire. The anonymity of the questionnaire was adopted to ensure the confidentiality of the participant's responses.

## Results

The current study assessed the awareness of women who are currently pregnant or have given birth in Jeddah towards complications of C-sections. A total of 507 eligible women participated in the study. Their ages ranged from 21 to 74 years old and most participants (36.9%) were in the age group of 32-42 years. Furthermore, the mean age of the study sample was 37.42 ± 9.7. The majority of participants, 429 (84.6%), were university graduates and the remaining had at least primary education. A total of 311 (61.3%) women were housewives and most participants, 296 (58.4%), had an income of more than 5000 riyals a month. Their demographic information is shown in Table 1.

**Table 1:** Sample distribution according to age group, level of education, Income, and job of the study participants (n=507)

Variables		Frequency	Percentage
Age group	21-31	167	32.9%
	32-42	187	36.9%
	43-53	123	24.3%
	54-74	30	5.9%
Education	Not educated	0	0.0%
	Primary school	5	1.0%
	Middle school	6	1.2%
	High school	67	13.2%
	University	429	84.6%
Employed	No	311	61.3%
	Yes	196	38.7%
Income	Less than 3000	123	24.3%
	3000-5000	88	17.4%
	More than 5000	296	58.4%

Table 2 shows the obstetric histories of the participants. The average number of pregnancies was 3.54 ± 2.1, and most women, 351 (69.2%), had one to four pregnancies each. Additionally, the mean number of deliveries was 2.9 ± 1.7; nearly all women, 462 (91.1%), had one to five deliveries. Also, most participants, 320 (63.1%), have never experienced abortion. As for their last mode of delivery, the majority of participants, 280 (55.2%), had spontaneous vaginal deliveries, and only 10 (2%) had instrumental deliveries. A total of 199 women (39.3%) delivered via C-sections in their last pregnancy. 

**Table 2:** Sample distribution according to gravidity, parity, abortion, and mode of last delivery (n=507)

Variables		Frequency	Percentage
Gravidity	1-4	351	69.2%
	5-8	147	29.0%
	>8	9	1.8%
Parity	0	13	2.6%
	1-5	462	91.1%
	6-10	32	6.3%
Abortion	0	320	63.1%
	1-3	180	35.5%
	>3	7	1.4%
Mode of Last Delivery	Not applicable	18	3.6%
	Spontaneous Vaginal Delivery	280	55.2%
	Cesarean -section	199	39.3%
	Instrumental Birth	10	2.0%

**Figure 1:**
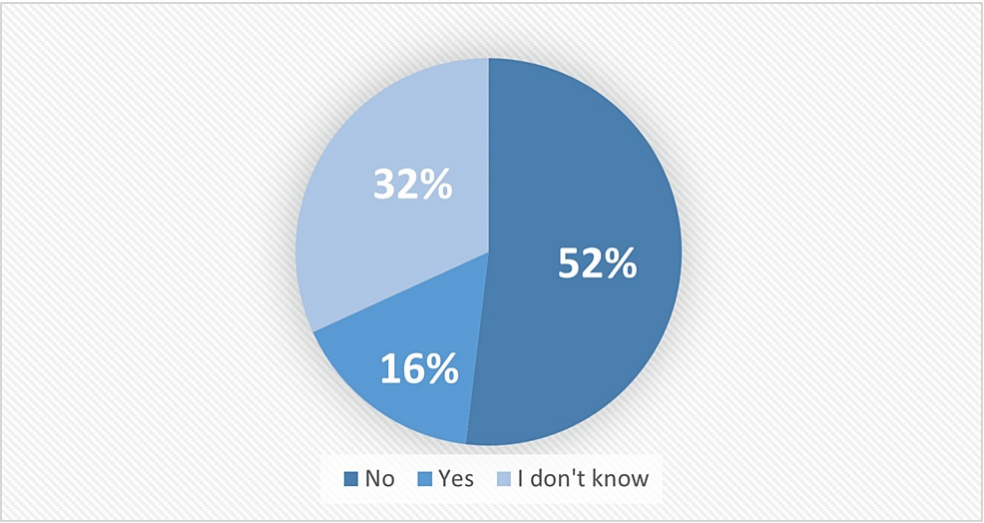
Do you think C-section is safer for your baby than spontaneous vaginal delivery? C-section: Caesarean section

Furthermore, 264 (52.1%) participants did not believe C-sections were safer for their babies, while 81 (16%) thought they were, as illustrated in Figure 1. A total of 393 (77.5%) women preferred vaginal births over Cesarean deliveries.

Of the 507 participants, 238 (46.9%) have experienced C-sections for different indications, and 98 (41.2%) had C-sections due to a history of multiple previous C-sections, making it the most common indication. At the same time, non-reassuring fetal monitoring was the second most common cause (79 (33.2%)). Moreover, the two most minor common indications were placenta previa and placenta accrete, with a rate of 5.5% and 2.9%, respectively. The rest of the indications for C-sections are listed in Table 3.

**Table 3:** Indication of C-section (n=238) C-section: Caesarean section

Questions (n=238)	Response	N	%
Life-threatening complication of pregnancy (pre-eclampsia- eclampsia, diabetes mellitus, cardiac diseases, respiratory diseases )	No	197	82.8%
	Yes	41	17.2%
Small maternal pelvis	No	175	73.5%
	Yes	63	26.5%
history of failed progress of labor	No	202	84.9%
	Yes	36	15.1%
Multiple pregnancies	No	213	89.5%
	Yes	25	10.5%
Abnormal fetal lie and presentation	No	167	70.2%
	Yes	71	29.8%
Placenta previa	No	225	94.5%
	Yes	13	5.5%
Placenta accrete	No	231	97.1%
	Yes	7	2.9%
Cesarean delivery on maternal request	No	201	84.5%
	Yes	37	15.5%
Non-reassuring fetal monitoring	No	159	66.8%
	Yes	79	33.2%
Other	No	180	75.6%
	Yes	58	24.4%

Finally, we evaluated the participants' knowledge about complications of C-sections, and the results obtained are listed in Table 4. At least 230 women (45.4%) had poor knowledge, 213 women (42%) had fair knowledge, and only 64 women (12.6%) had good knowledge, as shown in Table 5.

**Table 4:** Knowledge of C-section complications C-section: Caesarean section

Questions	Response	N	%
Hypoxia	No	337	66.5%
	Yes	170	33.5%
Need long time for recovery	No	67	13.2%
	Yes	440	86.8%
Hemorrhage	No	229	45.2%
	Yes	278	54.8%
	No	0	0.0%
Damage of internal organs and decrease in intestinal activity	No	306	60.4%
	Yes	201	39.6%
Infection of the wound, abdominal or urinary tract	No	241	47.5%
	Yes	266	52.5%
Venous thromboembolism	No	289	57.0%
	Yes	218	43.0%
Increase the rate of breathing problems and respiratory distress	No	355	70.0%
	Yes	152	30.0%
Rupture of the uterus	No	329	64.9%
	Yes	178	35.1%
Adhesion	No	194	38.3%
	Yes	313	61.7%
Abnormal placental implantations, placenta previa, placenta accrete	No	381	75.1%
	Yes	126	24.9%
Ectopic pregnancies	No	420	82.8%
	Yes	87	17.2%
Infertility	No	441	87.0%
	Yes	66	13.0%
Increased maternal and neonatal morbidity and mortality	No	381	75.1%
	Yes	126	24.9%
Loss the ability of normal vaginal delivery in future pregnancies	No	129	25.4%
	Yes	378	74.6%
It can lead to hysterectomy in some situation	No	333	65.7%
	Yes	174	34.3%

**Table 5:** Knowledge score

	Count	Percent %
Poor Knowledge	230	45.4
Fair Knowledge	213	42.0
Good Knowledge	64	12.6

The mean score was 6.26± 3.7. For most women, 60 (11.8 %), got a score of 5, while 21 (4.1%) got a maximum score of 15, and 22 women (4.3%) got a minimum score of 0. With regards to the complications of C-sections, prolonged recovery time was the complication that the majority of women, 440 (86.8%), were aware of; 378 participants (74.6%) knew that the possible inability to have standard vaginal delivery in the following pregnancies is an expected complication and 313 (61.7%) knew about adhesion. The least commonly known complications by the women included abnormal placental implantations and increased maternal and neonatal morbidity and mortality (126 (24.9%)), ectopic pregnancies (87 (17.2%)), and infertility (66 (13%)). The researchers made multiple statistical associations between the factors of the study, several of which were associations with the knowledge level of the participants. The first statistically significant relationship detected was between the age group and the participant's level of knowledge regarding C-sections (P = 0.030). Another statistically significant difference was found between the level of education and knowledge score (P = 0.026). Also, our results show that 82.2% of women with a university degree have poor knowledge, 42.9% have fair knowledge, and only 13.1% have good knowledge. In addition, there was a significant association between the level of knowledge and experiencing CDMR (P = 0.029). At least 64.9% of women who underwent CDMR had a poor understanding of C-section complications. Furthermore, a significant association was noted between the number of pregnancies and the knowledge score (P = 0.003), which showed that the higher the number of pregnancies, the greater the mother's awareness. Additionally, no significant association between income and knowledge score was observed in this study (P = 0.237).

Finally, the most statistically significant association was between the preferred mode of delivery and whether or not the participants' believed C-sections were safer for their babies (P = 0.001). Of the participants who preferred C-section as a mode of delivery, 43.9% believed it safer for their babies than vaginal delivery. 

## Discussion

This study aimed to assess the awareness of women towards complications of C-sections. Regarding the results of knowledge of the participants about complications of C-sections, most (45.4%) had poor knowledge, and only 12.6% had good knowledge. In contrast, in a study by Afaya et al., which was conducted in Ghana, a minority of participants (20%) had poor knowledge about C-sections [26]. The different knowledge scales and types of study questions may be the reason for the discrepancy between the two findings. Also, no significant difference in the knowledge score was observed between women who experienced C-sections and those who did not. These results were inconsistent with the findings of Jahromi et al., which showed that women who experienced C-sections had a lower mean average score of knowledge about their indications and complications than those who only experienced vaginal delivery [27]. On the contrary, the study by Afaya et al. revealed that previous experiences with C-sections increased the women’s knowledge about the procedure [26].

In this study, 44.1% of women with a university education had poor knowledge about C-section complications. Hypothetically, this is not surprising because a statically significant difference was observed in this relationship. Women with a university education were the most crucial proportion that filled out the questionnaire, and therefore their percentage is the highest. This outcome might be explained by the fact that several of the complications included in the questionnaire were uncommon and challenging to learn about. Contradictory results were obtained from the study conducted in Jeddah by Al Sulamy et al., where most participants were university-educated and had good knowledge of C-sections [28]. These results could be because of the nature of their questions, as they inquired about some of the most commonly known aspects of C-sections, so it was relatively easier to get a good score. Maternal age and the likelihood of experiencing C-section difficulties are significantly correlated. The age range of 32-42 years had the highest knowledge score, which may be because this group is still in their reproductive years and has most likely gone through more than one pregnancy, giving them greater exposure and more knowledge.

We also found a significant correlation between the number of pregnancies and the knowledge score, indicating that the more pregnancies a woman have, the more aware she is. This correlation may be explained by her increased experience and antenatal visits, during which her healthcare providers would explain delivery options and other relevant information. Similar results were obtained from the study by Afaya et al. [26]. Moreover, this study concluded that most women who opted for CDMR had poor knowledge about its complications. On the other hand, a 2019 study in Riyadh found that women with more medical knowledge about C-sections were 13.7 times more likely to request them without a medical necessity. This finding may be related to the fact that women with more medical knowledge about the procedure felt safer and more confident about the process [12]. No association between income and knowledge score was detected in this study, which is contrary to an Ethiopian study that revealed that the higher the income of the participants, the greater the awareness regarding C-sections [29]. The difference may further be linked with their level of education, and thus the higher the income, the higher the education level.

Prolonged recovery time was the most commonnly known complication, with 86.8% of the consequences the participants being aware of it. Our results were similar to those of Al Sulamy et al., in which most participants knew that prolonged hospital stay was a consequence of C-sections (85.4%) [28]. On the other hand, in this study, only about half (54.8%) of participants were aware of hemorrhages as a common complication. In comparison, in the survey by Ashimi et al., the possibility of blood transfusion was the most common complication that women were aware of (86.4%). Similarly, 90.3% of the Al Sulamy et al. study participants were aware of the possibility of blood transfusions [28]. In the present study, 74.6% of women knew that they may lose the ability to deliver through the vagina after a C-section, which corresponds with the results obtained by Jahromi et al., which showed that 63.5% were aware of the loss of the ability of natural delivery as a consequence of Cesarean delivery [27]. The complication least known by our participants was infertility (13%). Likewise, the Ethiopian 2017 study showed that of the people who were not willing to undergo C-sections, only 5.7% did so due to possible subsequent infertility [29]. The second most minor complication our participants were aware of was the possibility of ectopic pregnancies after C-sections, of which only 17.2% knew. Conversely, 70.3% knew about the risk of ectopic pregnancies in the Iranian study by Jahromi et al. [27]. The common complications vary from country to country, depending on educational levels, culture, customs, and traditions. Some common complications may be unknown due to lack of experience or education.

Various studies have shown that the rate of C-sections is increasing worldwide, while the WHO reported that the prevalence of C-sections shouldn’t exceed 10-15% [3,30]. The bulk of C-sections in the current study (46.9%) exceeds the recommended rate. A study done in India by Kumawat et al. reported that the prevalence of C-sections increased from 27.5% in 2010 to 39.6% in 2017 [31]. Another study in Iran by Rashidian et al. found that 50.12% of deliveries were Cesarean during the second half of 2017 [32]. In addition, multiple studies mentioned possible causes of increased C-section rates. Doctors contribute to choosing the delivery method, and many choose C-sections as they are more convenient and profitable because of their high cost, prolonged hospital stay, and drug use [33]. Also, a study done by Alehagen et al. reported that nowadays, C-sections are not only performed for the safety of the mother and fetus but are also considered luxurious by some communities and are requested even in the absence of medical indication [34]. Another study by Ahmed et al. reported that fear of labour pain is an essential factor that plays a role in the increasing prevalence of C-sections [12]. There are many other causes for the increased C-section rate, including the average increase in maternal age and the maternal obesity rate [35]. A study in the UK reported that delivery via C-section has three times greater risk for both mother and fetus than vaginal birth [36].

Regarding the indications for C-section delivery, the most common indication in the present study was previous C-section (19%), followed by non-reassuring fetal monitoring (15.6%) and abnormal fetal lie and presentation (14%). Similar results were found by Lunia et al. in an Indian study, which reported that most pregnant women who delivered by C-sections did so due to a previous history of the procedure (35.73%). Non-reassuring fetal monitoring came next with 19.64%, followed by abnormal fetal lie and presentation (9.45%) [35]. Another study conducted in Qassim by AlSheeha et al. reported that the most common indications were repeated Cesarean deliveries (21.5%), failure of labour progression (9.3%), and non-reassuring fetal monitoring (7.7%) [13]. In contrast, a study conducted in Ethiopia showed that the most common indication is fetal distress [37]. This difference could be attributed to fetal stethoscopes in the Ethiopian hospital where the study was performed, which were not proper electronic fetal monitoring systems. Most nurses and physicians were not trained to use a stethoscope, and the heartbeat sounds may be exaggerated since the measurement is subjective [37]. In the present study, maternal requests contributed to 7.3% of C-section indications. A study done in Riyadh by Ahmed et al. assessed the prevalence of CDMR, which was found to be 13.7% due to possible complications of vaginal birth (60%) and fear of labour pain and childbirth (46%) [12]. Moreover, when women had a previous single C-section, they usually chose a second C-section due to anxiety and fear of vaginal birth complications [38].

Regarding their preferred delivery method, most participants in our study preferred vaginal delivery. Kirane et al. conducted a study with similar results in which 81% chose vaginal delivery and only 13% preferred C-sections [39]. In general, the reason behind this consensus may reflect that although the participants of the different studies come from various cultures and beliefs, all of them are eventually encouraged to deliver vaginally rather than via C-sections. In addition, fear of being engaged in surgical procedures such as C-sections also play an essential role in their attitude and, thereby, their preferred mode of delivery.

Our study concluded that there was a significant relationship between the preferred mode of delivery and whether or not the participants believed C-sections were safer for their babies. Most women (61.7%) who thought C-sections were safer chose it as their preferred mode of delivery. Similar results were obtained from the study by Ibrahim et al., in which a higher proportion of females that delivered via C-section agreed that it was easier, safer, and more convenient [40].

One limitation of our atudy was that it used online surveys despite our best efforts to do personal intrviews, which couldn't be possible due to the coronavirus disease 2019 (COVID-19) pandemic and the mandatory measures. Also, in general, cross-sectional studies are limited by time, and recall bias might also exist. The knowledge level might be affected by the nature of our sample, which included a more significant number of women who gave birth by vaginal delivery.

## Conclusions

This study investigated the awareness of women in the antenatal and postnatal period in Jeddah, Saudi Arabia, regarding C-section complications. It concluded that most women had poor general knowledge about the possible difficulties, especially potential infertility and ectopic pregnancies that may follow a Cesarean delivery. However, most participants were well aware of the prolonged recovery time associated with the procedure and the possible loss of the ability to deliver vaginally in the subsequent pregnancy. The most common indication for Cesarean delivery for the women who experienced it was the history of previous C-sections. Surprisingly, the majority of women with university education had poor knowledge. Most women had a negative attitude toward C-sections and preferred vaginal deliveries. We recommend a question about the knowledge sources the women used to obtain information regarding C-sections, which may assist in analyzing the results presented by other research papers.

Furthermore, interviewing the participants in antenatal clinics would help future studies to achieve better results. Healthcare workers, including physicians, nurses, and midwives, should provide the necessary knowledge about possible C-section complications to pregnant women visiting antenatal clinics. They must be provided with alternative information sources such as leaflets and videos to better expand their knowledge, thus guiding them towards choosing the proper mode of delivery. The findings from this study will add to the existing literature and can be used to plan strategies to educate pregnant women about the benefits, risks, and complications of C-sections. At the same time, it also serves as a source of information to support further studies.
